# Genome Sequence of *Mycoplasma columbinum* Strain SF7

**DOI:** 10.1128/genomeA.00157-13

**Published:** 2013-04-18

**Authors:** Zisheng Guo, Xiaolong Xu, Qian Zheng, Tingting Li, Shichang Kuang, Zongde Zhang, Yushan Chen, Xidong Lu, Rui Zhou, Dingren Bi, Hui Jin

**Affiliations:** College of Veterinary Medicine, Huazhong Agricultural University, Wuhan, People’s Republic of Chinaa;; Division of Animal Infectious Disease in the State Key Laboratory of Agricultural Microbiology, Wuhan, People’s Republic of Chinab;; Animal Epidemic Prevention and Control Center of Hubei Province, Wuhan, People’s Republic of Chinac

## Abstract

*Mycoplasma columbinum* is a member of nonglycolytic *Mycoplasma* species which can hydrolyze arginine. Increasingly research has revealed that *M. columbinum* is associated with respiratory disease of pigeons and that the respiratory disease symptoms could be eliminated via the use of mycoplasma treatment medicine. Here we report the genome sequence of *M. columbinum* strain SF7, which is the first genome report for *M. columbinum.*

## GENOME ANNOUNCEMENT

*Mycoplasma columbinum* can be isolated from the trachea, oropharynx, air sac, and lung of healthy pigeons or those with mild symptoms of respiratory disease, including abnormal respiratory sounds, nasal discharge, sneezing, and difficulty breathing (1–3). It has been reported that *M. columbinum* was isolated from China, Britain, Japan, Nigeria, and other countries (1, 4, 5). We determined the genome of *M. columbinum* strain SF7, which was isolated by Bi et al. from oral and pharyngeal swabs of an apparently healthy pigeon in the Wuhan Province of China in 1997 (4).

The whole-genome sequencing of *M. columbinum* was performed by the Genome Sequencer FLX titanium platform (6) (Shanghai, China) and resulted in a total of 98,924 high-quality filtered reads with a total of 36.7 Mb and 49-fold coverage. The genome reads were assembled by using Newbler software of the 454 suite package into a total of 15 contigs (≥500 bp) with an average contig length of 49,880 bp. Putative protein-coding sequences were determined by the results of Glimmer 3.02 (7), Genemark, and Z-Curve programs. Tandem repeats (TR) were found by Tandem repeats finder with default parameters, and the TR average copy number was 3.6. Transfer RNA genes were identified by tRNAscan-SE (8). The ribosomal RNA genes were predicted by RNAmmer (9) and homology alignment through aligning the reference rRNA sequences to the assembled contigs. Functional annotation of the genes was performed by searching the NCBI nonredundant (nr) protein database as well as the KEGG (10) protein database, and COG (11) assignment was performed by RPS-BLAST using the NCBI CDD library. The metabolic pathways were constructed by using the KEGG database.

The genome of *M. columbinum* is composed of 748,210 bp with an average GC content of 26.95% and a coding percentage of 89.08%. There are 614 putative open reading frames (ORFs) with an average length of 1,089 bp, 32 tandem repeats, 8 pseudogenes, 2 rRNA operons, and 32 tRNA genes. The pathway of protein secretion of *M. columbinum* consists of SecY, SecD/F, SecA, and YidC, and the signal recognition particle pathway contains FfH and FtsY.

*M. columbinum* is a kind of arginine-utilizing nonglycolytic species, and the results of the biochemical tests confirmed that *M. columbinum* SF7 could not utilize glucose and hydrolyze arginine (4). Correspondingly, analysis of the *M. columbinum* SF7 genome revealed that the Embden-Meyerhof-Parnas pathway was incomplete but that the arginine dihydrolase pathway was found intact; the latter was comprised of arginine deiminase (MCSF7_00744), ornithine carbamoyltransferase (MCSF7_00739), and carbamate kinase (MCSF7_00734), encoded by *arcA*, *arcB*, and *arcC*, respectively, enough to support the ability of this organism to utilize arginine (12). The arginine dihydrolase pathway contributed to the energy supply, as reported previously, revealing that arginine could be hydrolyzed by this metabolic pathway to generate energy for arginine-utilizing *Mycoplasma* species (13, 14). All of these findings suggested that arginine was the major energy source for the arginine-utilizing species *M. columbinum* SF7.

In summary, this first-reported genome sequence of the *M. columbinum* species will be helpful for further research to elucidate the molecular mechanisms of metabolism and provide an unambiguous genetic background for confirming the etiological status.

### Nucleotide sequence accession numbers. 

The genome sequence of *Mycoplasma columbinum* strain SF7 was deposited at DDBJ/EMBL/GenBank and assigned accession number AFXA00000000. The version described in this article is the first version, AFXA01000000.
